# Impact of different automated peritoneal dialysis modalities on the inflammatory profile of elderly patients with chronic kidney disease

**DOI:** 10.1590/2175-8239-JBN-2022-0005en

**Published:** 2022-06-13

**Authors:** Marcia Maria Muniz de Queiroz Studart, Audrey Cecília Tonet Furioso, Joel Paulo Russomano Veiga, Mario Ernesto Rodrigues, Lucy de Oliveira Gomes, Clayton Franco Moraes

**Affiliations:** 1 Universidade Católica de Brasília, Gerontologia, Brasília, DF, Brasil.; 2 Universidade de Brasília, Ciências da Saúde, Brasília, DF, Brasil.; 3 Clínica Renal Care, Brasília, DF, Brasil.

**Keywords:** Chronic Kidney Disease, Automated Peritoneal Dialysis, Intermittent Nocturnal Peritoneal Dialysis, Continuous Cycling Peritoneal Dialysis, Inflammation in Peritoneal Dialysis, Inflammation in the elderly, Doença Renal Crônica, Diálise Peritoneal Automatizada, Diálise Peritoneal Intermitente Noturna, Diálise Peritoneal Contínua por Cicladora, Inflamação na Diálise Peritoneal, Inflamação em idosos

## Abstract

**Introduction:**

Chronic kidney disease, more prevalent in the elderly, is considered a public health issue worldwide.

**Objective:**

To evaluate the impact of automated, peritoneal dialysis modalities, intermittent and continuous, on the inflammatory profile of elderly people with chronic kidney disease.

**Methods:**

Prospective, cross-sectional and analytical study carried out in a dialysis clinic in Brasília - Brazil, with 74 elderly people aged 60 years or older. The patients underwent rapid Peritoneal Equilibration Test, clinical assessment, blood collection for biochemical and cytokine assessments, interleukin 6 and transforming growth factor beta 1, and answered a quality-of-life questionnaire (KDQOL-SF36). We used a 5% significance level for data analysis, associations and correlations.

**Results:**

Patients in the continuous modality had higher serum values of transforming growth factor beta 1 than those in the intermittent modality, which had higher peritoneal transforming growth factor beta 1, age and residual renal function than those in continuous mode. Interleukin 6 dosage in the peritoneum was associated with age, while serum IL-6 was associated with IL-6 in the peritoneum, time on dialysis and age. There was no association between the modality and the presence of diabetes, blood volume or nutritional status. Both modalities enable good adaptation to the dialysis treatment.

**Conclusion:**

Inflammation in automated peritoneal dialysis is mainly associated with low residual renal function, advanced age and longer time on therapy, and not to the type of dialysis performed.

## Resumo

**Introdução:**

A doença renal crônica, mais prevalente em idosos, é considerada um problema de saúde pública em todo o mundo.

**Objetivo:**

Avaliar o impacto das modalidades de diálise peritoneal automatizada, intermitente e contínua, no perfil inflamatório de idosos renais crônicos.

**Métodos:**

Estudo prospectivo, transversal e analítico realizado em uma clínica de diálise em Brasília, com 74 idosos com idade igual ou maior que 60 anos. Os pacientes foram submetidos ao Teste de Equilíbrio Peritoneal rápido, avaliação clínica, coleta de sangue para avaliações bioquímicas e de citocinas, interleucina 6 e fator de crescimento transformador beta 1, e questionário de qualidade de vida (KDQOL-SF36). Foram utilizadas para análise dos dados, associações e correlações com nível de significância de 5%.

**Resultados:**

Pacientes na modalidade contínua apresentaram valores séricos do fator de crescimento transformador beta 1 maiores do que os em modalidade intermitente. Estes apresentaram fator de crescimento transformador beta 1 no peritônio, idade e função renal residual maiores do que os em modalidade contínua. A dosagem da interleucina 6 no peritônio foi associada à idade, enquanto a IL-6 sérica foi associada à IL-6 no peritônio, ao tempo em diálise e à idade. Não houve associação entre a modalidade e a presença de diabetes, volemia ou estado nutricional. Ambas as modalidades permitem boa adequação à terapia dialítica.

**Conclusão:**

A inflamação na diálise peritoneal automatizada está associada principalmente à baixa função renal residual, à idade avançada e ao maior tempo em terapia, e não à modalidade de diálise realizada.

## Introduction

Chronic kidney disease (CKD), considered a public health issue worldwide^1^, is associated with poor quality of life and increased mortality at all ages^2,3^. Diabetes and arterial hypertension are its main causes^4,5,6^.

CKD is more frequent in the elderly, although it can affect individuals of all ages^1,4^, and its prevalence varies from 23% to 36% in people over 65 years of age^7^. Its incidence has been increasing significantly in the elderly population, reaching 20% of the population over 60 years of age and more than 40% of those over 80 years of age in Spain^2^, a similar prevalence among octogenarians in Brazil^4^, where a prevalence of 35% was detected in 2018, corresponding to about 46 thousand senior citizens^8^.

The increase in the number of CKD cases has been globally reported in recent decades^5,9^, as well as in the number of patients on dialysis^10^, justified by the aging and demographic transition of the population, resulting from the increase in life expectancy and the rapid process of urbanization^11^ . Although CKD affects individuals of all ages, it is more prevalent among the elderly worldwide^1,4^. The choice of renal replacement therapy (RRT) modality for the elderly should be shared^1,5^. Issues related to factors such as functional and cognitive status, comorbidities, frailty, disabilities and quality of life, in addition to medical indication, should be considered when choosing dialysis therapy^1,12,13^.

The presence of hemodynamic instability in hemodialysis is frequent, in addition to problems related to vascular access, especially in the elderly and diabetics, which has made peritoneal dialysis (PD) an increasingly indicated modality in these cases^14^, especially for the elderly, who may use lower doses of dialysis^15^, maintain more meaningful relationships with family members and greater freedom in therapy^5^.

PD is divided into two modalities: Continuous Ambulatory Peritoneal Dialysis (CAPD) and Automated Peritoneal Dialysis (APD), performed in different ways. CAPD is performed manually by the caregiver or patient. The APD modality is performed using a “cycling” machine, which infuses and drains the fluid into the patient’s peritoneal cavity in an automatic, cyclic and pre-programmed manner. APD is divided into Intermittent Nocturnal Peritoneal Dialysis (NIPD) and Continuous Cycling Peritoneal Dialysis (CCPD). In the latter, the patient has liquid in the peritoneal cavity 24 hours a day, undergoing continuous dialysis^16^. APD is the main PD modality in Brazil^8^, and its prevalence has been increasing worldwide^17,18^.

The systemic inflammation detected in PD patients is directly related to higher cardiovascular and general morbidity and mortality^19,20^; being associated with chronic cardiovascular, metabolic and nutritional adverse events, such as accelerated atherosclerosis, vascular calcification, sarcopenia, anorexia and resistance to erythropoietin^17^, consequently implicated in the increase in general morbidity and mortality in this population^19,20^.

The degree of peritoneal inflammation is directly related to the higher rate of complications from PD, as well as the lower survival of this dialysis method^21,22^, manifesting itself as an increase in the rate of peritoneal transport, failure of the technique and an increase in overall mortality^21^.

The causes of inflammation in PD patients are low or absence of residual renal function (RRF)^17^; hypervolemia^23^; high levels of serum endotoxins - probably secondary to intestinal bacterial translocation^14,23^; peritoneal aggression by bioincompatible PD solutions^17,18,20^; and production of inflammatory cytokines by adipose tissue^23^.

The conventional PD solution, the only one available in the SUS, is characterized by being acidic, having a high concentration of glucose, high osmolarity and having glucose degradation products (GDPs), being considered a bioincompatible solution, leading to peritoneal injury and toxicity^18,20^. Generated by the heat sterilization process, GDPs cause direct damage to mesothelial cells, leading to structural and functional changes, oxidative stress and limiting their ability to repair. The interaction between GDPs, advanced glycosylation end products (AGE) and AGE receptors can activate the intracellular signaling cascade and ultimately increase the expression of vascular endothelial growth factor (VEGF) and transforming growth factor beta (TGF)-beta)^20^. All these associated factors contribute to the development of submesothelial neoangiogenesis, vasculopathy and peritoneal fibrosis^10^.

No articles were found comparing the two regimens of automated, intermittent nocturnal and continuous dialysis in terms of inflammation, quality of life, dialysis adequacy, treatment survival, and overall mortality. Therefore, it is necessary to investigate whether there are differences between these two modalities of automated dialysis, to then offer the elderly a treatment with less inflammation and good adequacy, providing a better quality of life and longer survival for the patients and the method.

This study aimed to evaluate the impact of different modalities of automated PD (CCPD and NIPD) on the inflammatory profile of elderly patients with CKD.

## Material and Methods

This is a prospective, cross-sectional and analytical study carried out at the Renal Care dialysis clinic, located in the city of Brasília, DF - Brazil. The clinic is an overarching care center for renal pathologies, with nephrologists, nutritionists, nurses, pharmacists and social workers, who provide scheduled monthly and on-demand appointments. Here we had 40 patients treated by hemodialysis and 230 in a home PD program, both in the manual modality (CAPD) and in the automated modality (DPA), reimbursed by the Public Healthcare System - SUS and by health insurance companies, being the largest service in Brasília in terms of number of patients on PD.

Elderly patients, aged 60 years or older, were recruited for APD, accounting for 110 patients. We included those patients in the NIPD and CCPD modalities, and in a PD program for less than five years. The exclusion criteria were patients with neoplasms; acute adverse events in the 60 days prior to data collection, such as infections, inflammation, hospitalization for any reason, brain or cardiovascular disease; use of antibiotics or immunosuppressants; CAPD modality; cognitive changes; and those who refused to sign the Free and Informed Consent Form (ICF).

In the end, we recruited 74 patients; 58 in the NIPD modality and 16 in the CCPD modality. The patients were initially approached by telephone, and we explained the study objectives, data collection procedures, risks and benefits. After acceptance, a face-to-face meeting took place at the clinic for detailed explanations about the study, signature of the informed consent, medical evaluation, completion of clinical and quality of life data forms, completion of the mini mental state exam, which also excluded some from the study those patients who screened positive for dementia, as well as collection of laboratory test samples.

The patients included in the study underwent assessment of peritoneal transport through the fast Peritoneal EquilibrationTest (fast-PET), a procedure performed by the same nurse in all patients. The peritoneal cavity of the patient was drained in the continuous mode since this procedure does not require the intermittent mode because the cavity was empty. 2000 mL of DP solution with 2.5% Dianeal® dextrose (Baxter) were infused for 10 minutes and maintained for four hours. After this period, the peritoneal cavity was drained for 20 minutes in the sitting and standing positions and the effluent volume was measured, glucose and creatinine were collected from the drained fluid and serum creatinine, all in the fourth hour, which made it possible to calculate the ratio between the dialysate creatinine and serum creatinine (D/PCr), which classifies peritoneal transport into slow, medium-slow, medium-fast and fast, as this relationship decreases, according to Baxter (2012)^24^ and Twardowski (1987)^25^. The test was performed, without prejudice to the results, in the morning or in the afternoon, depending on the availability of the patients and the selected nurse.

At the end of the fast-PET, we collected the dialysate and blood samples to measure the inflammatory cytokines interleukin 6 (IL-6) and transforming growth factor beta 1 (TGF-beta 1), through the enzyme immunosorbent assay (ELISA) - by Booster®. Blood was also collected for biochemical assessment of dialysis adequacy and inflammation: blood count, potassium, phosphorus, serum albumin, C-reactive protein (CRP), urea, creatinine and alkaline reserve. This evaluation was performed using standard laboratory techniques with automatic analysis. All blood and peritoneal fluid samples for inflammatory cytokine measurements were taken and analyzed at the Immunogerontological laboratory of the Catholic University of Brasília (UCB). The blood samples for the other analyzes were sent to a clinical analysis laboratory.

The patients’ residual renal function (RRF) was measured through the mean of urea and creatinine clearances in the 24-hour urine collection, which was performed in the same week, on a different day from the fast-PET. The samples were sent for analysis to the same clinical analysis laboratory.

The principal investigator performed a clinical assessment of each patient on the day of the fast-PET to obtain clinical data, such as CKD etiology, time on PD, presence of comorbidities, uremic symptoms and medications in use, in addition to physical examination, with measurement of weight, height, blood pressure, pulmonary auscultation and evaluation of edema. A specific questionnaire on quality of life in chronic renal patients (KDQOL-SF36)^26^ was applied as one of the criteria for assessing the dialysis adequacy.

The results were divided into descriptive, association and correlation analysis. In the descriptive analysis, the qualitative variables were presented by means of frequency and percentage. The descriptive measures used for the quantitative variables were mean, median, standard deviation, minimum, maximum and interquartile range.

In the association analysis, inflammatory cytokines were compared between groups of PD, PET, edema, age and diabetes modalities using the Mann-Whitney U test, for variables with two categories, or using the Kruskal-Wallis test, for variables with three or more categories. Nonparametric tests were used, considering that no inflammatory cytokine, age, time on PD and RRF were normally distributed using the Kolmogorov Smirnov test (the normal distribution of data was rejected at a significance level of 5%).

In the correlation analysis, nonparametric tests were performed to assess the correlation of inflammatory cytokines with each other and between quantitative variables. The test used was the Spearman’s rho, which evaluates the correlation between the positions of the values of each variable. Assessments were performed for all patients and for patients belonging to each PD modality (NIPD and CCPD).

Data analyzes were performed using the IBM SPSS program (Statistical Package for the Social Sciences) 23, 2015. The significance level used was 5%.

## Results

We had 74 patients participating in the study. The mean age of the patients was 67.18±6.65 years, 90.5% were between 60 and 75 years old, 60.8% were men and 67.6% had diabetes (Table 1).

**Table 1:** Descriptive analysis of 74 elderly patients with chronic kidney disease in automated peritoneal dialysis in the renal care clinic, brasília, 2021

Variables		Mean	Standard deviation
**Age**		67.18	6.65
		n	%
**Sex**	**Females**	29	39.2
	**Males**	45	60.8
**Diabetes**	**Yes**	50	67.6
	**No**	24	32.4
**Age range**	**60 - 75 years**	67	90.5
	**Older than 75 years**	7	9.5

The elderly in the CCPD modality were significantly younger and had no RRF, or had minimal RRF, compared to those in NIPD, who had significantly higher RRF (Table 2).

Inflammatory cytokines were compared between the PD, PET, edema, age, and diabetes modality groups (Tables 2 and 3). Regarding the PD modality, serum TGF beta 1, peritoneal TGF beta 1, age and RRF were significantly associated. Patients undergoing CCPD had significantly higher serum TGF beta 1 values compared to those undergoing NIPD. On the other hand, the patients who underwent NIPD had significantly higher TGF beta 1 values in the peritoneum, age and RRF when compared with those who underwent CCPD (Table 2).

**Table 2:** Analysis of association between PD modalities, pet classifications, age, diabetes and inflammatory cytokines in 74 elderly people with chronic kidney disease in automated peritoneal dialysis the renal care clinic, brasilia, 2021

	PD Modality			PET			Age			Diabetes		
	NIPD	CCPD	P*	Low and medium	Medium high and high	P*	60-75 years	>75 years	P*	Yes	No	P*
Serum TGF beta 1 (pg/mL)	3.17 (3.89)	12.06 (5.89)	<0.001	4.51 (10.56)	3.84 (6.33)	0.041	4.06 (8.88)	6.73 (5.33)	0.314	4.06 (8.44)	4.51 (9.78)	0.66
Peritoneum TGF beta 1 (pg/mL)	4.78 (8.70)	1.30 (4.78)	0.039	4.34 (8.27)	4.34 (6.74)	0.559	4.78 (6.96)	0.43 (6.52)	0.212	3.91 (6.52)	4.78 (9.35)	0.342
Serum IL 6 (pg/mL)	0.00 (0.66)	0.00 (36.16)	0.242	0.00 (1.33)	0.00 (0.91)	0.837	0.00 (0.88)	0.60 (1.91)	0.34	0.00 (0.51)	0.00 (1.98)	0.358
Peritoneum IL 6 (pg/mL)	1.88 (22.21)	3.75 (18.67)	0.907	2.34 (7.96)	1.48 (30.96)	0.369	0.34 (18.21)	26.17 (32.08)	0.043	2.52 (22.00)	0.17 (20.92)	0.576
Age	67.00 (12.00)	61.50 (5.00)	0.018									
Time in PD	20.00 (25.50)	24.00 (34.50)	0.126									
RRF	6.52 (7.36)	0.50 (3.00)	<0.001									
PCR	0.4 (1.03)	1.31 (2.21)	0.077									

**Table 3:** Association between inflamatory citokines and pd modality with edema in 74 elderly patients with chronic kidney disease in autometed peritoneal dialysis at the renal care clinic, 2021

Variables		Edema						
		No edema		With edema		*P* *		
		Median	IR	Median	IR			
Serum TGF beta 1 (pg/mL)	4.51	8.33	3.62	8.43	0.183			
Peritoneal TGF beta 1 (pg/mL)	3.91	6.20	5.22	9.89	0.333			
Serum IL 6 (pg/mL)	0.00	1.24	0.00	1.24	0.736			
Peritoneal IL 6 (pg/mL)	0.76	24.54	3.33	12.72	0.986			
		N	%	N	%	*P* **	OR	CI95%
PD modality	NIPD	29	50.00	29	50.00	0.025	4.333	1.116 - 16.830
	CCPD	13	81.25	3	18.75			

* Mann-Whitney U test; IR = interquartile range

** Pearson`s chi-square test; N = number; OD = odds ratio; CI = confidence interval

As for PET classification, serum TGF beta 1 was significantly associated with PET. The patients classified as slow and medium-slow transporters had significantly higher serum TGF beta 1 values compared to the patients classified as fast and medium-fast transporters. No cytokine was significantly associated with the presence of diabetes or the presence of edema. IL 6 in the peritoneum was significantly associated with age. Older patients (> 75 years) had significantly higher IL 6 values in the peritoneum than younger patients (60 - 75 years) (Tables 2 and 3).

Serum IL 6 was positively correlated with IL 6 in the peritoneum in both PD modalities, NIPD and CCPD. This means that higher serum IL 6 values were significantly associated with higher IL 6 values in the peritoneum of elderly patients undergoing automated chronic peritoneal dialysis (Table 4).

**Table 4:** Correlation among inflammatory citokines from 74 elderly with chronic kidney disease in autometed peritoneal dialysis in the general modality (nipd plus ccpd), nipd and ccpd, at the renal care clinic - Brasília - Brazil, 2021

			Peritoneal TGF beta 1 (pg/mL)	Serum IL 6 (pg/mL)	Peritoneal IL 6 (pg/mL)
General (NIPD + CCPD)	Serum TGF beta 1 (pg/mL)	*P*	-0.197	0.171	0.073
		Coefficient	0.093	0.146	0.537
		n	74	74	74
	Peritoneal TGF beta 1 (pg/mL)	*P*		-0.032	-0.050
		Coefficient		0.788	0.672
		n		74	74
	Serum IL 6 (pg/mL)	*P*			0.639
		Coefficient			<0.001
		n			74
NIPD	Serum TGF beta 1 (pg/mL)	*P*	-0.106	0.097	0.167
		Coefficient	0.427	0.467	0.211
		n	58	58	58
	Peritoneal TGF beta 1 (pg/mL)	*P*		0.107	0.005
		Coefficient		0.423	0.968
		n		58	58
	Serum IL 6 (pg/mL)	*P*			0.681
		Coefficient			<0.001
		n			58
CCPD	Serum TGF beta 1 (pg/mL)	*P*	0.476	0.006	-0.251
		Coefficient	0.062	0.983	0.349
		n	16	16	16
	Peritoneal TGF beta 1 (pg/mL)	*P*		-0.361	-0.346
		Coefficient		0.169	0.189
		n		16	16
	Serum IL 6 (pg/mL)	*P*			0.508
		Coefficient			0.045
		n			16

* Spearman’s Ro

Regarding the other quantitative variables, serum IL 6 was positively correlated with time on PD, that is, higher levels of serum IL 6 were significantly associated with longer time on PD and age, being significantly higher in patients over 75 years of age in a chronic PD program. In addition, TGF beta 1 in the peritoneum was negatively correlated with alkaline reserve, and higher values of TGF beta 1 in the peritoneum were significantly correlated with the lowest concentration of bicarbonate in the blood of patients in an automated PD program, in both modalities. There was no difference regarding the nutritional status of the patients in both modalities, evaluated using the serum albumin value (Table 5).

**Table 5:** Correlation among inflammatory citokines and quantitative variables from 74 elderly with chronic kidney disease in autometed peritoneal dialysis in the general modalities (nipd plus ccpd), nipd (n=58) and ccpd (n=16), at the renal care clinic, Brasília - Brazil, 2021

			RRF	K	P	Albumin	Time in PD	Age	PCR	Alkaline Reserve
General (NIPD + CCPD)	Serum TGF beta 1 (pg/mL)	*P*	-0.139	0.156	0.171	0.015	0.155	-0.049	-0.060	0.048
		Coefficient	0.237	0.184	0.146	0.902	0.187	0.678	0.612	0.683
								
	Peritoneal TGF beta 1 (pg/mL)	*P*	-0.086	0.150	0.057	0.030	0.005	-0.085	-0.093	-0.241
		Coefficient	0.469	0.203	0.630	0.802	0.967	0.469	0.429	0.038
								
	Serum IL 6 (pg/mL)	*P*	-0.069	0.076	-0.010	-0.120	0.351	0.130	0.062	-0.022
		Coefficient	0.557	0.521	0.930	0.309	0.002	0.269	0.602	0.851
								
	Peritoneal IL 6 (pg/mL)	*P*	0.010	-0.028	-0.021	-0.039	0.213	0.174	0.197	-0.078
		Coefficient	0.935	0.810	0.861	0.742	0.069	0.138	0.092	0.510
								
NIPD	Serum TGF beta 1 (pg/mL)	*P*	0.185	0.093	-0.107	0.000	0.031	0.125	-0.127	0.018
		Coefficient	0.164	0.485	0.422	1.000	0.815	0.351	0.342	0.895
								
	Peritoneal TGF beta 1 (pg/mL)	*P*	-0.282	0.132	0.161	-0.055	0.046	-0.112	0.007	-0.324
		Coefficient	0.032	0.322	0.227	0.679	0.733	0.402	0.959	0.013
								
	Serum IL 6 (pg/mL)	*P*	0.047	0.005	-0.121	-0.133	0.314	0.274	0.000	0.012
		Coefficient	0.727	0.973	0.365	0.319	0.016	0.038	0.997	0.931
								
	Peritoneal IL 6 (pg/mL)	*P*	0.085	-0.050	0.010	-0.040	0.179	0.305	0.155	-0.016
		Coefficient	0.527	0.712	0.941	0.767	0.180	0.020	0.244	0.907
								
CCPD	Serum TGF beta 1 (pg/mL)	*P*	0.032	0.615	0.018	0.236	0.191	-0.155	-0.529	0.204
		Coefficient	0.908	0.011	0.948	0.380	0.479	0.566	0.035	0.450
								
	Peritoneal TGF beta 1 (pg/mL)	*P*	0.264	0.370	0.206	0.264	0.038	-0.338	-0.267	-0.135
		Coefficient	0.324	0.159	0.443	0.323	0.890	0.200	0.317	0.619
								
	Serum IL 6 (pg/mL)	*P*	-0.253	0.212	0.101	-0.110	0.414	-0.093	0.005	0.019
		Coefficient	0.344	0.430	0.710	0.686	0.111	0.732	0.985	0.943
								
	Peritoneal IL 6 (pg/mL)	*P*	-0.360	0.097	-0.401	0.040	0.274	-0.303	0.273	-0.298
		Coefficient	0.171	0.720	0.124	0.883	0.304	0.254	0.306	0.262

* Spearman’s Ro

In the NIPD modality, TGF beta 1 in the peritoneum was negatively correlated with RRF, that is, higher values of TGF beta 1 in the peritoneum were significantly associated with low RRF in patients on a chronic PD program in this modality (Table 5).

In the CCPD modality, serum TGF beta 1 was positively correlated with serum potassium (K) levels. Thus, higher levels of serum TGF beta 1 were significantly associated with higher levels of potassium (K) in patients on a chronic PD program in this modality. Serum TGF beta 1 was negatively correlated with CRP, and higher serum TGF beta 1 values were significantly associated with lower CRP values, with no significant association between CRP and IL6 (Table 5).

Regarding the assessment of general quality of life and quality of life in dialysis therapy, using the KDQOL-SF 36 questionnaire, one of the parameters suggested to assess adequacy in PD, patients on NIPD presented general health assessment, dialysis therapy on their life and emotional well-being significantly superior to those of CCPD patients. While the latter had significantly higher rates of satisfaction with the therapy and perceived greater encouragement from the medical team compared to those in NIPD.

## Discussion

There is an association between RRF reduction or loss and increased inflammation, both in pre-dialysis and dialysis patients^17^, in addition to higher rates of anemia, malnutrition, hypoalbuminemia and serum levels of CRP^18^.

In this study, older adults in the CCPD modality were significantly younger and had no or minimal RRF compared to those in NIPD, who showed higher residual renal function. The finding of significantly higher serum TGF beta 1 levels in patients in the CCPD modality can be attributed to the lower RRF, also found significant in this group, which leads to lower renal clearance of medium-size molecules, since peritoneal clearance is very low, a fact previously reported in several studies^14,20^.

Michels et al. (2011)^27^ found greater loss of RRF in patients on APD compared to those on CAPD, which was not confirmed in other studies, so there is no consensus so far as to the impact of the PD modality on RRF18, since one of the main indications for choosing continuous modalities is the absence of RRF^28^.

It is believed that TGF-beta, especially TGF-beta 1, is the key mediator of peritoneal fibrosis, leading to fibroblast activation and collagen deposition in the extracellular matrix, in addition to epithelial-mesenchymal transition^10,14,15,21^. TGF-beta 1 in the peritoneal fluid is associated with the type of peritoneal transport and it induces the production of vascular endothelial growth factor (VEGF), a cytokine responsible for neoangiogenesis, proving that fibrosis and neovascularization occur simultaneously in the peritoneal membrane during the inflammatory process^10^.

Yu et al. (2019)^22^ detected high rates of peritoneal transport in patients with high levels of IL 6 in the peritoneal fluid, in disagreement with the present study, in which no significant association was detected between faster PET and greater peritoneal inflammation. Lambie et al. (2013)^29^, in agreement with our findings, did not find this association. Zhou et al. (2015)^21^ also found no association between PET and systemic inflammation, in agreement with what was found in the present study. These findings can be attributed to the short and equivalent time on PD in both modalities, on average 22 months, to the fact that only a single patient of each modality was using hypertonic solutions (dextrose at 4.25%), and to the irrelevant rate of peritonitis episodes in both groups (only one patient from each modality), factors classically associated with fast transport PET and the presence of peritoneal inflammation^17,30,31^.

Over the years of PD, the peritoneal membrane undergoes structural and functional changes, especially in mesothelial cells, increasing the risk of peritonitis and loss of this membrane, with ultrafiltration (UF) failure^20,21,32^. IL 6 is considered a central mediator in the intraperitoneal inflammatory response and its levels in the peritoneal fluid increase with time in PD^21^. This finding agrees with the finding of the present study, in which serum IL6 and peritoneal fluid IL6 were positively correlated with longer PD time in both modalities.

Research has suggested that intermittent PD modalities reduce damage to the peritoneal membrane caused by inflammation^20^ and improve the negative impact caused by the type of rapid peritoneal transport^15^, the latter directly associated with the level of intraperitoneal inflammation^22^. On the other hand, Levin et al. (2006)^29^, in the classic KDOQI guideline of the National Kidney Foundation, suggest that continuous PD modalities should be preferred to intermittent modalities, aiming at a greater clearing of medium molecules, such as inflammatory cytokines. In the present study, there was no significant association between serum IL 6 and peritoneal IL 6 measurements with the two PD modalities investigated. On the other hand, the significant association found in this study between higher serum TGF beta 1 levels in CCPD patients and higher peritoneal fluid TGF beta 1 levels in those on NIPD can be attributed to low RRF and older age in these groups, respectively.

According to Kooman et al. (2017)^23^, during the aging process, several proteins are damaged as a result of non-enzymatic glycosylation, generating advanced glycosylation end-products (AGEs) and leading to increased membrane permeability, as it occurs in PD patients, leading to increased intraperitoneal inflammation with increasing age. Similar significant findings were found in this study, in which serum and peritoneal IL 6 were positively related to age, with higher values being found in patients over 75 years of age compared to those aged between 60 and 75 years. Also, the significant finding of higher levels of TGF beta 1 in the peritoneum of NIPD patients may be associated with the older age of this group of patients.

According to Wang et al. (2020), we estimate that the prevalence of volume overload in patients undergoing PD treatment ranges from 27% to 66.8%. Excess body fluid in dialysis patients is also considered a factor associated with increased systemic inflammation, being a common finding and associated with increased mortality from all causes and from cardiovascular diseases^33,34^. However, there was no significant association between the levels of inflammatory cytokines and the presence of clinically identified edema in the present study in any of the modalities.

Regarding the assessment of adequacy in PD, recently recommended criteria were used to define good adequacy in PD, such as: absence of edema, presence of RRF, adequate control of serum levels of potassium, phosphorus and albumin, in addition to good quality of life on dialysis^30,35,36^. In the present study, both modalities were able to provide adequate therapy to patients, as the presence of edema was significantly more frequent in the NIPD group, even though this group showed a significantly higher RRF, which can be attributed to the greater freedom of water intake, lower time per day in therapy and, consequently, lower ultrafiltration, in addition to the presence of dialysis-free days in this modality, with no significant differences regarding potassium, phosphorus and albumin levels between the modalities. Regarding quality-of-life assessment, patients of both modalities showed significant quality of life requirements on dialysis, assessed by questionnaires classically used for this purpose in this population, KDQOL-SF-36^37^.

## Conclusion

Inflammation in automated peritoneal dialysis in the elderly is mainly associated with low residual renal function, advanced age, and longer time on therapy, rather than with the type of dialysis performed, either intermittently or continuously.
